# Impact of Methicillin-Resistant *Staphylococcus aureus* Nasal Screening in Lower Respiratory Tract Infections: A Systematic Review Incorporating Network and Bayesian Meta-Analyses

**DOI:** 10.1093/ofid/ofag178

**Published:** 2026-03-27

**Authors:** Tristan T Timbrook, Zijie Zhang, Tamara Krekel

**Affiliations:** Department of Pharmacy, Barnes-Jewish Hospital, Saint Louis, Missouri, USA; Department of Pharmacotherapy, University of Utah College of Pharmacy, Salt Lake City, Utah, USA; Department of Pharmacotherapy, University of North Carolina at Chapel Hill Eshelman School of Pharmacy, North Carolina, USA; Department of Pharmacy, Barnes-Jewish Hospital, Saint Louis, Missouri, USA

**Keywords:** antibiotic duration, antimicrobial stewardship, lower respiratory infection, meta-analysis

## Abstract

**Background:**

Methicillin-resistant *Staphylococcus aureus* (MRSA) pneumonia requires empirical therapy coverage for patients with risk factors, yet MRSA causes <1% of community-acquired and 20%–40% of nosocomial pneumonias. Methicillin-resistant *Staphylococcus aureus* polymerase chain reaction (PCR) nasal screening offers rapid results with 95%–99% negative predictive value. We systematically evaluated clinical outcomes associated with MRSA nasal screening in hospitalized patients with lower respiratory tract infections (LRTIs).

**Methods:**

We searched PubMed and EMBASE through 29 June 2024 for studies evaluating MRSA nasal screening in adult inpatients with LRTIs. The primary outcome was duration of MRSA therapy. Secondary outcomes included frequency of vancomycin trough monitoring, hospital length of stay, incidence of acute kidney injury (AKI), in-hospital mortality, and 30-day readmission. We performed frequentist and Bayesian meta-analyses.

**Results:**

Fifteen studies (2872 patients) were included. Pharmacist-driven protocols (PDP) with PCR versus standard of care significantly reduced MRSA therapy duration (mean difference −1.34 days, 95% CI −1.59 to −1.08; *I*^2^ = 61%). Network meta-analysis showed PDP+PCR was most effective (−1.51 days versus no testing, 95% CI −2.04 to −.98), while PCR or culture alone showed no significant benefit. PDP+PCR reduced vancomycin trough monitoring (OR 0.21, 95% CI .13–.33), AKI (OR 0.54, 95% CI .39–.76), and in-hospital mortality (OR 0.70, 95% CI .50 to .96). Bayesian analysis showed >99% probability of exceeding minimal clinically important differences for duration of MRSA therapy and trough monitoring, but 36% or less for other outcomes.

**Conclusions:**

Methicillin-resistant *Staphylococcus aureus* nasal PCR screening with pharmacist-driven protocols significantly reduces MRSA therapy exposure and improves clinical outcomes in hospitalized LRTI patients, supporting broader implementation of this diagnostic-antimicrobial stewardship synergy.

## Teaser

Methicillin-resistant *Staphylococcus aureus* (MRSA) nasal testing combined with pharmacist-driven protocols reduced MRSA therapy duration by 1.34 days and decreased the odds of vancomycin trough monitoring by 79% in hospitalized LRTI patients. Bayesian analysis showed >99% probability of benefit, supporting broader implementation.

## BACKGROUND

Methicillin-resistant *Staphylococcus aureus* (MRSA) pneumonia carries substantial morbidity and mortality, with community-acquired MRSA pneumonia demonstrating mortality rates approaching 30%, which is approximately 3 times higher than other pneumonia etiologies [1, 2]. In hospital-acquired pneumonia (HAP) and ventilator-associated pneumonia (VAP), MRSA mortality reaches 55% [3]. This severity necessitates prompt empirical coverage, usually with vancomycin, when MRSA is suspected based on risk factors. However, MRSA accounts for less than 1% of community-acquired pneumonia (CAP) cases [4] and 20%–40% of nosocomial pneumonia cases in the United States [5]. This epidemiology creates a clinical dilemma of balancing the imperative for effective empirical coverage against the risks of unnecessary antimicrobial exposure, adverse events, and healthcare costs.

Traditional respiratory culture results require up to 96 hours for processing, delaying targeted therapy decisions. Additionally, up to 64% of CAP patients are unable to produce a sputum specimen and the test is often low yield when produced [6]. The MRSA nasal polymerase chain reaction (PCR) swab has emerged as a rapid diagnostic tool to guide empirical vancomycin use; given a specificity of 90.3% and low prevalence of MRSA pneumonia, the MRSA nasal PCR demonstrates a negative predictive value (NPV) of 95%–99% for ruling out MRSA pneumonia [7]. This high NPV enables clinicians to safely discontinue or withhold empirical MRSA therapy in PCR-negative patients, potentially reducing antimicrobial exposure, adverse events, and healthcare costs.

Recognizing this diagnostic utility, the 2019 Infectious Diseases Society of America (IDSA) CAP Guidelines endorsed routine MRSA nasal PCR testing for patients with MRSA risk factors or those already receiving empirical MRSA therapy [8]. The 2024 IDSA CAP Update Pathway further reinforced this recommendation [9]. However, limited outcomes data supported the initial recommendation, and no systematic evaluation has comprehensively assessed the clinical impact of MRSA nasal PCR testing in hospitalized patients with suspected or confirmed lower respiratory tract infections (LRTIs).

This study aimed to evaluate the process measures and clinical outcomes associated with MRSA nasal screening in patients with suspected or confirmed LRTI.

## METHODS

### Literature Review

We searched and reviewed PubMed and EMBASE from inception to 29 June 2024 for studies in English evaluating the clinical outcomes after MRSA PCR nasal swab was added for guiding the de-escalation or discontinuation of empirical MRSA therapy. We used the following search query: (methicillin resistant *Staphylococcus aureus* or MRSA) AND (nasal OR nares) AND (polymerase chain reaction OR PCR) AND (swab OR screening OR assay) AND (pneumonia OR respiratory OR lower respiratory tract infections). Two authors searched literature, performing a first pass review of the titles and abstracts for relevance. Then a second pass review was performed on the full text of all potentially relevant studies. Differences in article selection were resolved by consensus. Hand searches of bibliographies of included studies were performed to identify additional studies of relevance.

### Inclusion and Exclusion Criteria

Studies were eligible if they reported clinical outcomes of hospitalized adult patients with suspected or confirmed LRTIs which included CAP, HAP, VAP, healthcare-associated pneumonia (HCAP), aspiration pneumonia, or chronic obstructive pulmonary disease (COPD) exacerbation. Intervention patients were required to undergo MRSA nasal or oropharyngeal screening by PCR or culture. Chronic obstructive pulmonary disease exacerbation and aspiration pneumonia were included due to the frequent diagnostic overlap and similar empiric antibiotic management in these syndromes. Studies were categorized by comparative design (eg, PCR vs no testing, PCR integrated into a pharmacist-driven protocol [PDP] vs PCR alone). Prognostic comparisons (eg, PCR positive vs negative result on outcomes) were also included. Non-English studies, studies without MRSA screening data, and studies lacking comparative clinical outcomes were excluded.

### Outcomes

The primary outcome selected was duration of MRSA therapy as measured in days. This served as the primary endpoint due to therapy changes being the most proximal nonperformance impact of a diagnostic testing that is routinely measured [10, 11]. Secondary outcomes included frequency of vancomycin trough monitoring, hospital length of stay (LOS), incidence of acute kidney injury (AKI), in-hospital mortality, 30-day readmission, and cost outcomes. Acute kidney injury was defined according to the criteria reported in the primary studies. Duration of therapy was defined based on days of therapy, calculated as the number of calendar days during which at least 1 dose of a medication was administered. Where available, cost measures were corrected for inflation using the country's Consumer Price Index as well as adjusted to a common currency.

### Data Extraction and Quality Assessment

Data extraction used a standardized form capturing study design, patient population, intervention characteristics, and outcomes. Two reviewers independently assessed study quality using Cochrane RoB 2 (Risk of Bias 2) tool for randomized trials and ROBINS-I (“Risk Of Bias In Non-randomized Studies—of Interventions”) tool for nonrandomized studies, resolving disagreements through discussion and consensus.

### Data Synthesis, Analysis, and Reporting

Meta-analyses were initially performed using frequentist methods with Cochrane's RevMan for producing forest plots. Frequentist methods were primarily employed given their widely used and accepted use in the medical community. For the primary pairwise analysis, “Standard of Care” (SOC) was defined as a composite comparator representing the baseline diagnostic strategy utilized in the control arm (eg, PCR alone, culture alone, or no routine screening). To simultaneously compare multiple interventions, we also conducted a frequentist network meta-analysis (NMA) using a random-effects model in the *netmeta* R package. This approach allowed for the integration of direct and indirect evidence across the treatment network. To ensure the reliability of the NMA, we used node-splitting analysis to check whether direct comparisons between treatments aligned with indirect evidence across the network.

Subsequently, Bayesian meta-analyses were performed to complement the frequentist approach. While frequentist methods provide parameter estimates and confidence intervals based on long-run frequency and sampling distributions, Bayesian methods directly estimate the posterior probability that treatment effects exceed clinically meaningful thresholds. This approach allows for probabilistic statements about clinical benefit (eg, the probability that MRSA therapy duration is reduced by more than 0.5 days).

Bayesian analyses were conducted using the *metafor* and *bayesmeta* packages in R. Two prior specifications were employed for each outcome: (1) weakly informative priors derived from relevant systematic reviews (Supplementary Table 2), and (2) noninformative priors to assess sensitivity to prior assumptions [12]. For heterogeneity parameters, we applied Turner's empirically derived priors based on Cochrane Database analyses [13]. When literature-based priors were unavailable for specific outcomes, only non-informative prior results were reported. This dual approach enables assessment of whether conclusions are robust to prior specification or primarily driven by the current data.

For Bayesian probabilistic determinations as clinical thresholds, we selected distribution-based thresholds over anchor-based methods for establishing minimal clinically important difference (MCID). Anchor-based MCIDs are expert consensus on the thresholds while distribution-based are derived from standardized statistical shifts in effect. Distribution-based MCIDs were chosen due to the lack of consensus in anchor-based approaches and their limited applicability to meta-analytic inference across the diverse endpoints in our review [14]. Distribution-based methods using Cohen's effect size conventions provide a standardized, replicable framework that enables consistent interpretation across diverse outcome measures while maintaining established precedent in clinical research [15]. We established MCID thresholds using a unified framework based on Cohen's standardized effect sizes (Supplementary Table 2). To ensure consistent interpretation across outcome types, we translated the standardized *d* = 0.3 threshold into corresponding odds ratio (OR) metrics for binary outcomes. We employed a probit model where the baseline risk is mapped to a standard normal distribution and shifted by the effect size (*d*) to derive a “flexible MCID.” This approach accounts for the baseline event rates observed in our evidence base, ensuring the OR thresholds are clinically interpretable and adjusted for the risk environment of each outcome. To visualize the clinical implications of our findings, we generated cumulative probability plots of the posterior distribution under both informative and non-informative priors. These plots depict the probability that the treatment effect exceeds each value on the x-axis, with the vertical line representing the MCID. This approach allows transparent assessment of the degree of certainty that the intervention achieves a clinically meaningful benefit as well as an assessment of alternative thresholds.

Random-effects models accounted for anticipated heterogeneity arising from behavioral clinician responses to diagnostics and stewardship interventions. Continuous outcomes were synthesized using mean differences, and binary outcomes using ORs or risk ratios. Medians and related dispersion measures (ie, interquartile range [IQR], range) data were converted to means and standard deviations (SDs) via the Box-Cox method using the *estmeansd* package in R [16]. When transformed estimates yielded implausible results (eg, means exceeding 75th percentiles, SD greater than twice the IQR), original methods from Luo et al and Wan et al were applied [17, 18]. Where missing, means or SDs were imputed using linear model prediction from the remainder of the studies [19]. Specifically, missing SDs were estimated by modeling the relationship between reported means and SDs within each outcome group. For studies with zero events in 1 arm, a standard continuity correction of 0.5 was applied to enable the calculation of odds ratios. Heterogeneity was assessed with *I*^2^ and Cochran Q tests. Publication bias was assessed with funnel plots. Certainty of findings was assessed narratively based on study design, risk of bias, heterogeneity, and consistency of results across included studies. Although we did not formally apply GRADE criteria, we assessed the certainty of evidence narratively based on the quality and precision of contributing data for each outcome.

Reporting adhered to PRISMA guidelines (Supplementary Table 1) and study was preregistered with PROSPERO (CRD420251102985).

## RESULTS

**Figure 1.**
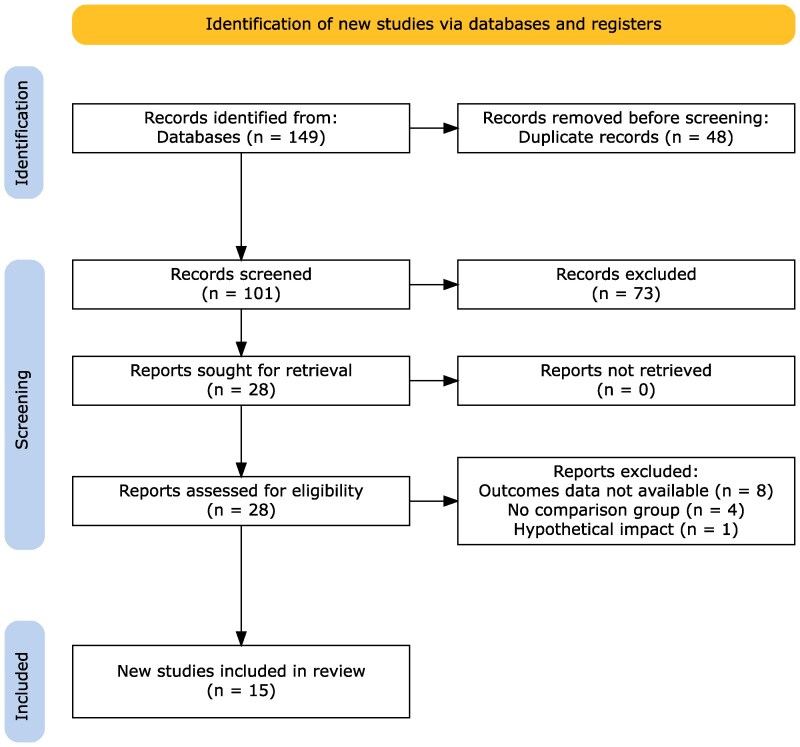
PRISMA flow diagram.

Our literature search resulted 149 reports meeting our search strategy, and after study selection, there was an inclusion of 15 studies with 2872 patients (Figure 1). Characteristics of the included studies are in Table 1. Most studies (71%) were published since 2020. All studies were conducted among adults and in the United States, with 50% of hospitals being reported as community hospital settings; all studies except 3 (21%) were quasi-experimental pre-post studies. Nine studies included patients on vancomycin and 6 included patients on vancomycin or linezolid. Median study sample size was 156 patients. Studies were heterogeneous in their LRTI inclusion, including various combinations of CAP, HAP, HCAP, VAP, aspiration pneumonia, general pneumonia, respiratory tract infection, and COPD exacerbations. A median of 37% of patients had ICU admissions among those reporting a proportion of ICU patients. To minimize confounding by indication on therapy duration, the majority of included studies (n = 11) explicitly excluded patients with concomitant bacteremia or extrapulmonary MRSA infections. The most common intervention evaluated was a pharmacist-driven protocol combined with PCR testing (PDP+PCR), which was most frequently compared against a SOC that utilized PCR testing without a formal protocol. Studies most commonly compared PDP+PCR to MRSA PCR alone (6 studies), followed by PDP+PCR to no MRSA screening (2 studies), PDP+PCR to MRSA screening culture alone (2 studies), MRSA PCR alone to no MRSA screening (2 studies), and 1 study included MRSA screening culture alone compared with no MRSA screening. One study included 3 phases: pre/post education phases alongside PDP+PCR as well as a baseline period before implementation.

**Table 1.** Characteristics of Included Studies in Systematic Review and Meta-Analysis

Study Author	Study Design	Setting	Population (Inclusion)^a^	Sample Size	Respiratory Infection Type	ICU (%)	Immunocompromised Type	Intervention	Comparison Group
Baby (2017) [20]	Retrospective cohort	Community teaching hospital	Adults on vancomycin or linezolid	57	CAP (12.3%)/HCAP (68.4%)/HAP (19.3%)	36.8%	Immunosuppression (31.6%)	PDP+PCR	No testing
Cho (2022) [21]	Retrospective cohort	Community hospital	Adults on vancomycin	77	PNA	100%	NR	PCR alone	No testing
Dadzie (2019) [22]	Retrospective cohort	Rural 235-bed hospital	Adults on vancomycin	79	PNA	0%	Immunosuppression (1.3%)	PDP+PCR	PCR alone
Diep (2021) [23]	Retrospective cohort	Academic medical center	Adults on vancomycin	418	CAP (43.3%)/HAP or VAP (56.7%)	100%	Immunocompromised (21.5%)	PDP+PCR	Culture alone
Doan (2024) [24]	Pre-post (3-phase)	Community teaching hospital	Adults on vancomycin or linezolid	110	CAP (67.3%)/HAP (32.7%)	0%	NR	Phase 2—PDP+PCR	Phase 1—PCR alone
Dunaway (2018) [25]	Pre-post	Teaching hospital	Adults on vancomycin	196	CAP (25%)/HCAP (75%)	15.3%	Chemotherapy (14.8%)/Immunosuppression (5.6%)	PDP+PCR	PCR alone
Evans (2021) [26]	Pre-post	Community teaching hospital	Adults on vancomycin	196	CAP (60.2%)/HAP (27%)/VAP (4.1%)/Aspiration (8.7%)	NR	NR	PDP+PCR	PCR alone
Huffman (2020) [27]	Pre-post	Community hospital	Adults on vancomycin	62	Respiratory tract infection	NR	NR	PDP+PCR	PCR alone
Marinucci (2023) [28]	Pre-post	Tertiary care community hospital	Adults on vancomycin or linezolid	100	CAP (36%)/HAP (64%)	100%	Immunocompromised (22%)	PDP+PCR	PCR alone
Meng (2021) [29]	Pre-post	Academic medical center	Adults on vancomycin	610	CAP (58.4%)/HAP (30.3%)/VAP (11.3%)	44.9%	Immunocompromised (38.5%)	PDP+PCR	Culture alone
Pham (2021) [30]	Pre-post	Community teaching hospital	Adults on vancomycin	210	CAP (5.7%)/HAP (22.8%)/HCAP (58.6%)/Aspiration (12.9%)	25.2%	NR	PDP+PCR	No testing
Renzoni (2021) [31]	Retrospective cohort	Academic medical center	Adults on vancomycin or linezolid	116	CAP (46.6%)/HAP (53.4%)	0%	NR	Culture alone	No testing
Rowe (2024) [32]	Retrospective cohort	Academic medical center	Adults with COVID-19; vancomycin or linezolid	293	CAP (97.3%), HAP (2%), VAP (<1%)	6.4%	NR	PCR+ vs PCR-	N/A
Sindelar (2022) [33]	Retrospective cohort	Academic medical center	Adults on vancomycin or linezolid	341	PNA	NR	Solid tumor (23.5%)/Leukemia (1.2%)/Lymphoma (5.6%)	PCR alone	No testing
Willis (2017) [34]	Pre-post	Tertiary care community hospital	Adults on vancomycin	300	HCAP/HAP/CAP/COPDE	0%	Immunocompromised (19.7%)/Chemotherapy or radiation (13.3%)/HIV-1 (4%)	PDP+PCR	PCR alone

Abbreviations: CAP, community-acquired pneumonia; COPDE, chronic obstructive pulmonary disease exacerbation; HAP, hospital-acquired pneumonia; HCAP, healthcare-associated pneumonia; N/A, not applicable; NR, not reported; PCR, polymerase chain reaction; PDP, pharmacist-driven protocol; PNA, pneumonia; VAP, ventilator-associated pneumonia.

^a^MRSA therapy was predominantly vancomycin; specific linezolid (LZD) counts included: Rowe (n = 65), Sindelar (n = 27), Doan (n = 3), Renzoni (n = 1), and Marinucci (n = 1). All other studies utilized vancomycin exclusively or did not delineate the specific split.

 

#### Methicillin-Resistant *Staphylococcus aureus* Therapy Duration

**Figure 2.**
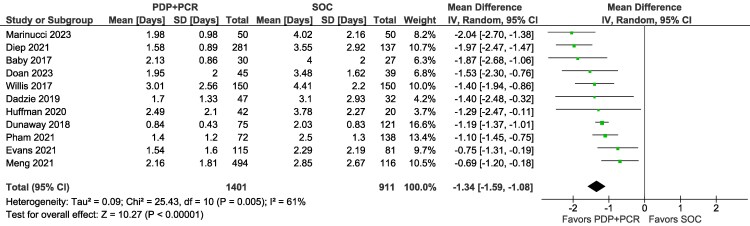
Days of MRSA therapy with PDP+PCR vs SOC in LRTI patients. Abbreviations: LRTI, lower respiratory tract infection; MRSA, methicillin-resistant *Staphylococcus aureus*; PCR, polymerase chain reaction; PDP, pharmacist-driven protocol; SOC, standard of care. Doan 2023 was a multi-phase implementation study analyzed from baseline to active intervention phase.

PDP+PCR versus SOC in LRTIs was associated with a reduced therapy duration of MRSA coverage. Among 11 studies with 2312 participants [20, 22–30, 34], the mean difference (MD) in duration of MRSA therapy was significantly lower with PDP+PCR (MD −1.34 days, 95% CI −1.59 to −1.08; *I*^2^ = 61%, Cochran's Q *P* = .005) than SOC comparisons (Figure 2). Bayesian meta-analysis demonstrates that PDP+PCR reduces therapy duration from a baseline of 3.14 days by a mean of 1.31 (95% CrI −1.61 to −1.03) days with noninformative priors and 1.57 (95% CrI −1.83 to −1.31) days with informative priors, with both models showing 100% probability of exceeding the 0.65-day MCID threshold (Supplementary Figure 1).

**Figure 3.**
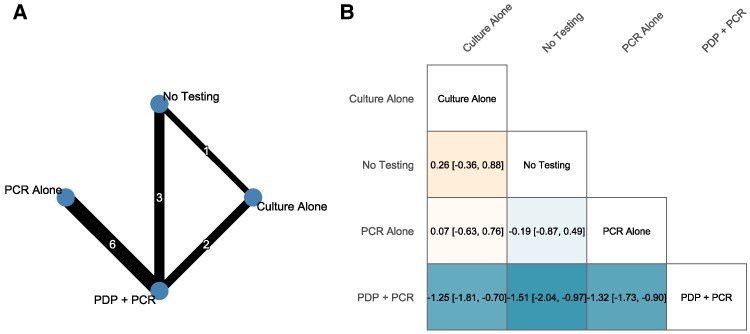
Network meta-analysis of MRSA nasal surveillance strategies on therapy duration. Abbreviations: MRSA, methicillin-resistant *Staphylococcus aureus*; PCR, polymerase chain reaction; PDP, pharmacist-driven protocol. *A*, shows the network plot where nodes represent intervention strategies and lines indicate direct comparisons (thickness proportional to the number of studies). *B*, displays the league table with mean differences in vancomycin duration (d) and 95% CIs. Values should be read as row treatment minus column treatment, with negative values favoring the row treatment (eg, PDP+PCR compared with No Testing shows −1.51 [−2.04, −0.97] d). The network includes 12 studies with 2421 participants.

NMA was performed for direct and indirect comparisons included among all available testing intervention studies excluding studies that conditioned upon unknowable results at the time of the testing intervention decision. Initial analysis of all 13 studies analyzed for NMA revealed significant global inconsistency (*Q* = 21.36, *P* < .05). Local inconsistency testing via node-splitting revealed no significant differences between direct and indirect evidence for any comparison (all *P* values > .05). Leave-one-out sensitivity analysis identified one 3-phase study [24] as the primary contributor to the inconsistency. Excluding this study resolved the inconsistency (*Q* = 2.62, *P* = .11) while maintaining similar treatment effect estimates. The primary analysis therefore included 12 studies with 12 treatment comparisons. Among the 12 studies with 2421 participants [20–23, 25–31, 34], a NMA comparing strategies to no testing found PDP+PCR was the most effective in reducing antibiotic duration (Figure 3). Compared with no testing, the NMA showed PCR alone (MD −0.19 days, 95% CI: −.87 to .49) and culture alone (MD 0.26 days, 95% CI −.36 to .88) were not significantly associated with MRSA therapy reduction, while PDP+PCR showed a statistically significant reduction in MRSA therapy (MD −1.51 days, 95% CI: −2.04 to −.97) and PCR with post-PDP phase interventions (*I*^2^ = 68.0%, Cochran's Q *P* = .002).

Among the individual results from 3 primary studies [27, 32, 33] comparing MRSA therapy duration by PCR results, a negative PCR result was associated with a significant decrease in duration with linezolid (3.5 vs 7 days, *P* = .0016) but not vancomycin (2 vs 3 days, *P* = .12) when compared with a positive PCR result, a 1.65-day decrease (mean 2.35 vs 4.01 days, *P* < .05) in MRSA therapy duration with a negative PCR with PDP+PCR versus a negative PCR with PCR alone, and a 0.67-day decrease in MRSA therapy duration with a negative PCR versus no screening testing (median 1 vs 2 days, *P* < .05).

#### Secondary Outcomes

**Figure 4.**
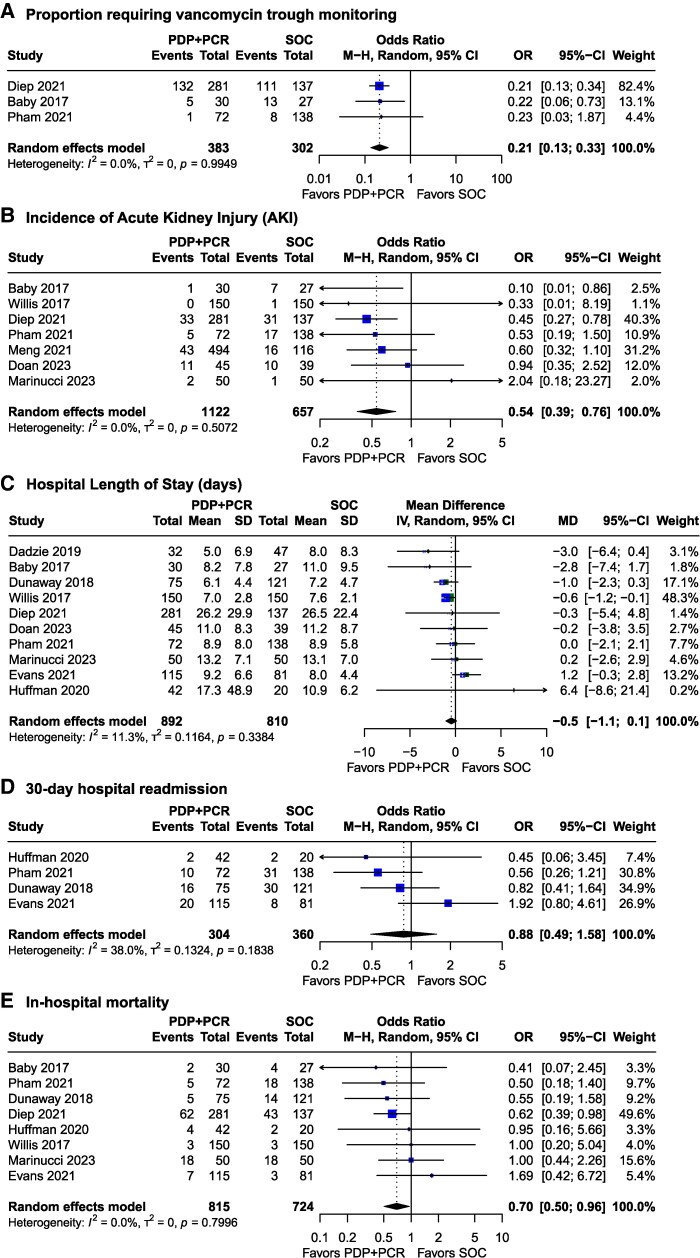
Secondary clinical and process outcomes associated with pharmacist-driven MRSA screening protocols (PDP+PCR) versus standard of care. Abbreviations: CI, confidence interval; M-H, Mantel–Haenszel; IV, inverse variance; df, degrees of freedom.

Secondary outcomes generally favored the PDP+PCR strategy compared with SOC. From a baseline of 43.7%, the proportion of patients receiving at least 1 vancomycin trough was significantly lower with PDP + PCR (OR 0.21, 95% CI .13–.33; *I*^2^ = 0%; Cochran's Q*P* = .99; Figure 4*A*). Bayesian analysis with an MCID of OR 0.58 showed >99% probability that PDP+PCR reduces trough monitoring compared with SOC (Supplementary Figure 2).

For patient safety outcomes, frequentist analysis showed statistically significant difference in AKI (OR 0.54, 95% CI .39–.76; *I*^2^ = 0%; Cochran's Q *P* = .51) (Figure 4*B*). From a baseline AKI incidence of 11.4%, Bayesian analysis with a non-informative prior suggested 35.5% probability of achieving clinically meaningful AKI reduction (MCID of OR 0.55) (Supplementary Figure 3). The informative prior, based on expected vancomycin exposure-toxicity relationships, yielded 0% probability of achieving the MCID.

Hospital LOS was not significantly shorter with PDP+PCR versus SOC strategies (MD −0.5 days, 95% CI −1.1 to .1; *I*^2^ = 11%, Cochran's Q *P* = .34) (Figure 4*C*). From a baseline LOS of 13.14 days, Bayesian analysis using a clinically-based MCID of 1-day reduction showed a 5.9% posterior probability that PDP+PCR achieves this threshold with noninformative priors, decreasing to 0.4% with informative priors (Supplementary Figure 4).

There was no significant difference in hospital 30-day readmission with PDP+PCR versus SOC strategies (OR 0.88, 95% CI .49 to 1.58; *I*^2^ = 38%, Cochran's Q *P* = .18) (Figure 4*D*). From a baseline readmission of 19.7%, Bayesian analysis using an MCID of OR 0.58 yielded posterior probabilities of 8.3% with noninformative priors and 0.1% with informative priors for achieving clinically meaningful readmission reduction with PDP+PCR (Supplementary Figure 5).

However, in-hospital mortality (OR 0.70, 95% CI .50 to .96; *I*^2^ = 0%, Cochran's Q *P* = .80) was significantly lower with PDP+PCR as compared with SOC (Figure 4*E*). A post hoc exploratory subgroup analysis of in-hospital mortality by care setting revealed no statistically significant differences among subgroups (ICU: OR 0.69, 95% CI .46 to 1.04; non-ICU: OR 1.00, 95% CI .20 to 5.04; mixed/not reported: OR 0.67, 95% CI .37 to 1.19; test for subgroup differences *P* = .90). With a baseline in-hospital mortality of 19.7%, Bayesian analysis using an MCID of OR 0.58 with a noninformative effect prior indicated a 8.3% posterior probability of reduced in-hospital mortality with PDP+PCR (Supplementary Figure 6) while an informative prior reflected 0.1%.

Limited economic data were reported across included studies [27, 29]. Two studies provided cost analyses with varying methodologies and completeness. Meng et al [29] reported the most comprehensive analysis, demonstrating a median cost avoidance of $40.33 per patient (estimated yearly total of $28 231), incorporating drug acquisition costs ($15 per 1 g vancomycin dose), laboratory costs ($37 per vancomycin level, $36 per PCR test), and personnel time based on Bureau of Labor Statistics wages. Huffman et al [27] found median cost savings of $23.61 per patient ($75.30 preintervention vs $51.69 postintervention, *P* < .01) using drug costs of $9/day for vancomycin at average wholesale price, plus laboratory costs of $20 per PCR test and $13 per vancomycin level. After adjusting for inflation to 2024U.S. dollars, the per-patient savings ranged from approximately $29 to $50.

#### Bias Assessments

Looking at the included studies, the risk of bias assessment reveals low risk across 71 of 98 (72%) individual domain evaluations, with the primary concern being potential serious confounding (D1) affecting 6 of 14 (43%) of studies (Supplementary Figure 7). All studies were rated as a moderate risk of bias in selection of reported results given the absence of preregistration. Regarding publication bias, the forest plots (Supplementary Figures 8–13) display the distribution of effect estimates across included studies. Visual inspection of the plots does not reveal obvious asymmetric patterns typically associated with publication bias, though the small number of studies precludes definitive assessment. Cumulatively, the reliance on non-randomized studies with the majority of a moderate risk of bias, combined with substantial heterogeneity for the primary outcome, places the overall certainty of evidence as moderate for the reduction in therapy duration and low to moderate for secondary outcomes.

## DISCUSSION

The use of MRSA nasal screens to guide de-escalation of empiric MRSA therapy demonstrated significant clinical benefits in this meta-analysis. We observed a 1.3-day reduction in therapy duration, 79% decreased odds of vancomycin trough monitoring, 46% decrease in odds of AKI, and 30% decrease in odds of in-hospital mortality, supporting current IDSA CAP guidelines recommending routine PCR MRSA nasal screening for managing empirical MRSA therapy in LRTIs. Bayesian probabilistic analyses using both informative and noninformative priors consistently showed high probabilities of benefit across multiple outcomes, including reduction in duration of MRSA therapy and reduced vancomycin trough monitoring, though other outcomes reflected a low probability of effectiveness with probabilities of 36% or less. Network meta-analysis reinforced that diagnostic testing alone is insufficient; only when combined with PDP did MRSA PCR consistently reduce MRSA therapy use. Importantly, the intervention showed no signal for harm, with no increase in 30-day readmissions or in-hospital mortality, highlighting its safety alongside its effectiveness.

A notable finding of this meta-analysis is the significant reduction in in-hospital mortality, an effect primarily localized to the ICU population (OR 0.69; 95% CI .46–1.04). This survival benefit is biologically plausible when linked to our observed 46% reduction in the odds of AKI. By utilizing a pharmacist-driven protocol to rapidly act on negative PCR results, clinicians spare high-risk patients the cumulative nephrotoxic exposure of unnecessary empirical vancomycin. These findings align with recent evidence suggesting that excessive vancomycin use, particularly when doses are prolonged beyond clinical necessity, is independently associated with increased mortality [35]. Thus, the synergy of rapid diagnostics and active stewardship functions not just as a duration-reduction tool, but as a critical safety intervention for the most vulnerable patients.

Our findings align with existing literature demonstrating the synergistic effect of rapid diagnostics combined with antimicrobial stewardship intervention. Similar patterns have been observed in bloodstream infections, where systematic reviews consistently show enhanced outcomes when rapid diagnostics are paired with active stewardship intervention [36, 37]. The unexpected magnitude of benefit from MRSA nasal screening, despite its relative simplicity compared with multiplex PCR panels, raises important questions about optimizing other underutilized diagnostics. While active interventions require substantial resources, clinical decision support systems have shown promise in randomized trials for improving respiratory illness management and enabling effective passive stewardship when integrated with rapid diagnostics; more research is needed to examine this approach. Given that all US hospitals now maintain antimicrobial stewardship programs, targeted active interventions using MRSA nasal screening in LRTI represents a high-value opportunity. Finally, in the absence of active interventions there appeared to be differences in impact with linezolid versus vancomycin for negative results. Given the overall small number of linezolid patients reported among studies herein, future research should further evaluate possible variations of effect by MRSA agent.

Despite limited economic data, the cost-effectiveness implications appear favorable. Studies showed cost savings of $29 to $50 while potentially yielding clinical benefits. However, the existing analyses lacked clarity regarding cost perspective (hospital vs patient), cost categories (direct vs indirect, fixed vs variable), and a comprehensive accounting of resource utilization. For example, intravenous therapy requires approximately 20 additional minutes of nursing time per dose compared with oral therapy and carries increased environmental costs through higher carbon footprints. Robust economic modeling incorporating full costs and clinical outcomes is needed to quantify the true value proposition.

This analysis has several strengths and limitations. We employed informative priors derived from systematic reviews of randomized trials for most outcomes, while also presenting noninformative prior analyses to allow unbiased data interpretation. We provided comprehensive probability distributions, enabling readers to apply their own clinical thresholds and prior beliefs. Notable limitations include the paucity of pediatric outcome studies and the absence of confounder adjustment in included studies which is a common shortcoming in diagnostic outcome research [36, 37]. Inconsistent reporting and overlapping categories in the primary data precluded standardization, which limits our ability to draw firm conclusions regarding the intervention's impact specifically within several subgroups (eg, immunocompromised, COPD, CAP vs non-CAP, mechanical ventilation). Individual patient data meta-analysis should be considered in the future to explore potential differences of effect in these patient populations. Additionally, our research did not contain patients not started on initial therapy for eligibility which limits conclusions on how positive test results may impact initiations of therapy—additional research should explore this area. Finally, the NMA was limited by few studies per comparison, potentially affecting the estimate precision.

In conclusion, MRSA nasal screening represents an effective strategy for optimizing MRSA therapy in hospitalized patients with respiratory infections, particularly when combined with PDP. These findings support broader implementation while highlighting the need for additional research in pediatric populations and economic outcomes.

## Supplementary Material

ofag178_Supplementary_Data

## Supplementary Data

Supplementary materials are available at *Open Forum Infectious Diseases* online. Consisting of data provided by the authors to benefit the reader, the posted materials are not copyedited and are the sole responsibility of the authors, so questions or comments should be addressed to the corresponding author.
