# Supplementary material for: Impact of Methicillin-Resistant Staphylococcus aureus Nasal Screening in Lower Respiratory Tract Infections: A Systematic Review Incorporating Network and Bayesian Meta-Analyses
Source: Open Forum Infect Dis. 2026 Mar 27;13(4):ofag178. doi: 10.1093/ofid/ofag178 (PMC13069566; doi:10.1093/ofid/ofag178)
Supplement: ofag178_Supplementary_Data [file ofag178_supplementary_data.docx]

**Supplementary Materials**

**Table S1.** PRISMA 2020 checklist

| **Section and Topic** | **Item #** | **Checklist item** | **Location where item is reported** |
| --- | --- | --- | --- |
| **TITLE** | | |  |
| Title | 1 | Identify the report as a systematic review. | Page 1 |
| **ABSTRACT** | | |  |
| Abstract | 2 | See the PRISMA 2020 for Abstracts checklist. | Page 2 |
| **INTRODUCTION** | | |  |
| Rationale | 3 | Describe the rationale for the review in the context of existing knowledge. | Page 3 |
| Objectives | 4 | Provide an explicit statement of the objective(s) or question(s) the review addresses. | Page 3 |
| **METHODS** | | |  |
| Eligibility criteria | 5 | Specify the inclusion and exclusion criteria for the review and how studies were grouped for the syntheses. | Pages 3-4 |
| Information sources | 6 | Specify all databases, registers, websites, organisations, reference lists and other sources searched or consulted to identify studies. Specify the date when each source was last searched or consulted. | Page 3 |
| Search strategy | 7 | Present the full search strategies for all databases, registers and websites, including any filters and limits used. | Page 3 |
| Selection process | 8 | Specify the methods used to decide whether a study met the inclusion criteria of the review, including how many reviewers screened each record and each report retrieved, whether they worked independently, and if applicable, details of automation tools used in the process. | Page 3 |
| Data collection process | 9 | Specify the methods used to collect data from reports, including how many reviewers collected data from each report, whether they worked independently, any processes for obtaining or confirming data from study investigators, and if applicable, details of automation tools used in the process. | Page 4 |
| Data items | 10a | List and define all outcomes for which data were sought. Specify whether all results that were compatible with each outcome domain in each study were sought (e.g. for all measures, time points, analyses), and if not, the methods used to decide which results to collect. | Page 4 |
|  | 10b | List and define all other variables for which data were sought (e.g. participant and intervention characteristics, funding sources). Describe any assumptions made about any missing or unclear information. | Page 4 |
| Study risk of bias assessment | 11 | Specify the methods used to assess risk of bias in the included studies, including details of the tool(s) used, how many reviewers assessed each study and whether they worked independently, and if applicable, details of automation tools used in the process. | Page 5 |
| Effect measures | 12 | Specify for each outcome the effect measure(s) (e.g. risk ratio, mean difference) used in the synthesis or presentation of results. | Page 5 |
| Synthesis methods | 13a | Describe the processes used to decide which studies were eligible for each synthesis (e.g. tabulating the study intervention characteristics and comparing against the planned groups for each synthesis (item #5)). | Pages 4-5 |
|  | 13b | Describe any methods required to prepare the data for presentation or synthesis, such as handling of missing summary statistics, or data conversions. | Pages 4-5 |
|  | 13c | Describe any methods used to tabulate or visually display results of individual studies and syntheses. | Pages 4-5 |
|  | 13d | Describe any methods used to synthesize results and provide a rationale for the choice(s). If meta-analysis was performed, describe the model(s), method(s) to identify the presence and extent of statistical heterogeneity, and software package(s) used. | Pages 4-5 |
|  | 13e | Describe any methods used to explore possible causes of heterogeneity among study results (e.g. subgroup analysis, meta-regression). | Pages 4-5 |
|  | 13f | Describe any sensitivity analyses conducted to assess robustness of the synthesized results. | Pages 4-5 |
| Reporting bias assessment | 14 | Describe any methods used to assess risk of bias due to missing results in a synthesis (arising from reporting biases). | Page 5 |
| Certainty assessment | 15 | Describe any methods used to assess certainty (or confidence) in the body of evidence for an outcome. | Page 5 |
| **RESULTS** | | |  |
| Study selection | 16a | Describe the results of the search and selection process, from the number of records identified in the search to the number of studies included in the review, ideally using a flow diagram. | Page 5, Figure 1 |
|  | 16b | Cite studies that might appear to meet the inclusion criteria, but which were excluded, and explain why they were excluded. | Figure 1 |
| Study characteristics | 17 | Cite each included study and present its characteristics. | Page 5, Table 1 |
| Risk of bias in studies | 18 | Present assessments of risk of bias for each included study. | Page 7, Figure S14-20 |
| Results of individual studies | 19 | For all outcomes, present, for each study: (a) summary statistics for each group (where appropriate) and (b) an effect estimate and its precision (e.g. confidence/credible interval), ideally using structured tables or plots. | Pages 5-7, Figures 2-3, Figures S2-S13 |
| Results of syntheses | 20a | For each synthesis, briefly summarise the characteristics and risk of bias among contributing studies. | Pages 5-7. Figure S14-20 |
|  | 20b | Present results of all statistical syntheses conducted. If meta-analysis was done, present for each the summary estimate and its precision (e.g. confidence/credible interval) and measures of statistical heterogeneity. If comparing groups, describe the direction of the effect. | Pages 5-7, Figures 2-3, Figures S2-S13 |
|  | 20c | Present results of all investigations of possible causes of heterogeneity among study results. | Page 6, Figure 3 |
|  | 20d | Present results of all sensitivity analyses conducted to assess the robustness of the synthesized results. | - |
| Reporting biases | 21 | Present assessments of risk of bias due to missing results (arising from reporting biases) for each synthesis assessed. | Page 7, Figure S14-20 |
| Certainty of evidence | 22 | Present assessments of certainty (or confidence) in the body of evidence for each outcome assessed. | Pages 5-7, Figures 2-3, Figures S2-S13 |
| **DISCUSSION** | | |  |
| Discussion | 23a | Provide a general interpretation of the results in the context of other evidence. | Pages 7-8 |
|  | 23b | Discuss any limitations of the evidence included in the review. | Pages 7-8 |
|  | 23c | Discuss any limitations of the review processes used. | Pages 7-8 |
|  | 23d | Discuss implications of the results for practice, policy, and future research. | Pages 7-8 |
| **OTHER INFORMATION** | | |  |
| Registration and protocol | 24a | Provide registration information for the review, including register name and registration number, or state that the review was not registered. | Page 3 |
|  | 24b | Indicate where the review protocol can be accessed, or state that a protocol was not prepared. | - |
|  | 24c | Describe and explain any amendments to information provided at registration or in the protocol. | - |
| Support | 25 | Describe sources of financial or non-financial support for the review, and the role of the funders or sponsors in the review. | Page 8 |
| Competing interests | 26 | Declare any competing interests of review authors. | Page 8 |
| Availability of data, code and other materials | 27 | Report which of the following are publicly available and where they can be found: template data collection forms; data extracted from included studies; data used for all analyses; analytic code; any other materials used in the review. | - |

**Table S2.** Literature selections for Bayesian priors

| Outcome | Prior Distribution | Source Effect Estimate | Notes on Prior Selection | Reference |
| --- | --- | --- | --- | --- |
| Heterogeneity Priors |  |  |  |  |
| In-hospital mortality | Log-Normal | Not Applicable | Prior for heterogeneity (τ²) based on a predictive distribution for a new random-effects meta-analysis derived from a large database of diverse interventions. | Turner et al. [13] |
| Resource use / hospital stay / process | Normal | Not Applicable | Prior for heterogeneity (τ²) based on a predictive distribution for a new random-effects meta-analysis derived from a large database of diverse interventions. | Turner et al. [13] |
| Adverse events | Log-Normal | Not Applicable | Prior for heterogeneity (τ²) based on a predictive distribution for a new random-effects meta-analysis derived from a large database of diverse interventions. | Turner et al. [13] |
| Effect Priors |  |  |  |  |
| Duration of therapy reduction (days) | Normal | -1.83 [-2.15, -1.50] | Prior on the mean difference. | Schultz et al. [38] |
| Vancomycin trough monitoring† | Not identified | Not identified | No suitable prior was identified in the literature. | Not identified |
| Acute kidney injury | Log-Normal | OR = 1.12 [1.02, 1.23] | Prior on the log-OR. Estimate is the adjusted odds ratio (aOR) for each additional treatment day (p=0.02). | Cano et al. [39] |
| In-hospital mortality | Log-Normal | OR = 0.83 [0.70, 0.99] | Prior on the log-OR. Estimate is the adjusted odds ratio (aOR) for procalcitonin-guided therapy vs. control (p=0.037). | Schultz et al. [38] |
| Length of stay (days) | Normal | -0.19 [-0.96, 0.58] | Prior on the mean difference (p=0.626). | Schultz et al. [38] |
| 30-day readmission | Log-Normal | OR = 1.13 [0.71, 1.78] | Prior on the log-OR. Estimate is the odds ratio for readmission (p=0.61; I²=0%). | Lin et al. [40] |

Note. For continuous outcomes, we selected Cohen’s d = 0.3 as the MCID, reflecting a moderate effect size that balances clinical relevance with statistical detectability. This choice is empirically supported by a systematic review of 107 HTA-funded superiority randomized controlled trials, which reported a median target standardized effect size of d = 0.3 [Rothwell. *Trials.* 2018]. To ensure consistent interpretation across outcome types, we translated this standardized threshold into corresponding metrics for binary outcomes. Specifically, we converted d = 0.3 to odds ratio (OR) thresholds using the probit method, conditional on baseline event rates observed in our evidence base, enabling clinically interpretable OR thresholds while maintaining consistency with the standardized MCID definition [Horita. *J Clin Que.* 2024]. While others used Cohen's d = 0.3 for MCID determination, hospital LOS MCID was set at 1 day reduction based on established healthcare economics literature demonstrating substantial cost savings (~$1,000-2,000 per day) and clinical benefits of earlier discharge [Golan.  *Ann Intern Med.* 2005].

**Figure S1.** Bayesian posterior cumulative probability distributions for reduction in MRSA therapy duration under informative and non-informative priors


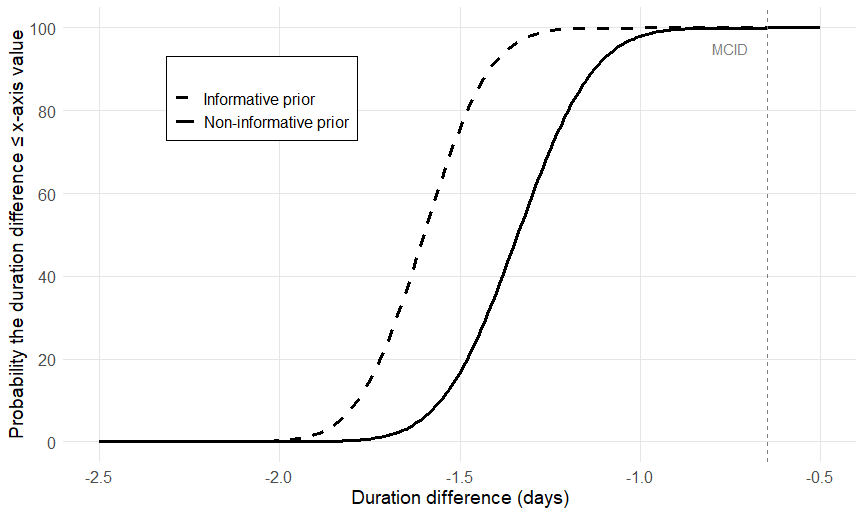


Abbreviations: MCID, minimal clinically important difference.

**Figure S2.** Bayesian cumulative probability of reduced vancomycin trough monitoring


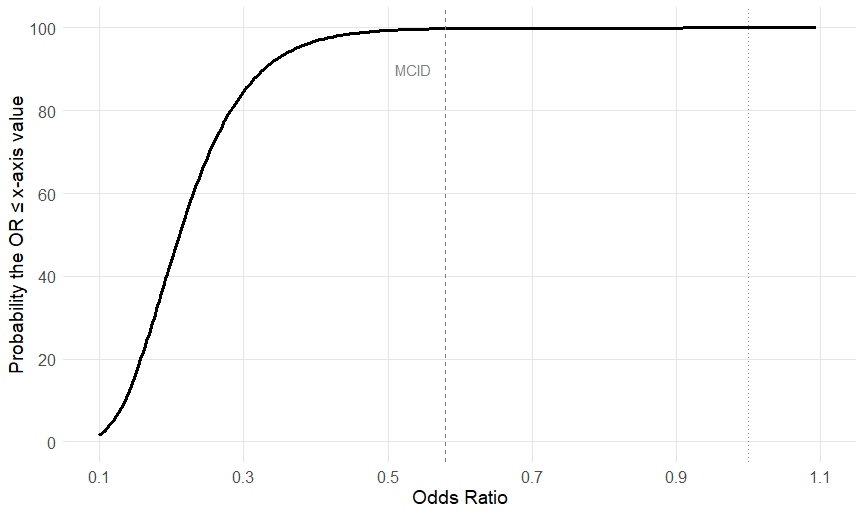


Abbreviations: MCID, minimal clinically important difference; OR, odds ratio.

**Figure S3.** Reduction in AKI cumulative density function of the probabilities plotted against the distribution of effects


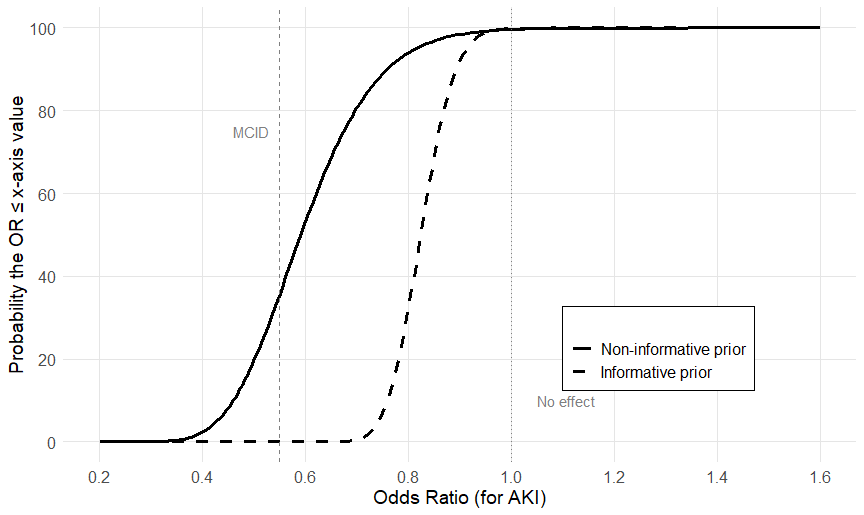


Abbreviations: AKI, acute kidney injury; MCID, minimal clinically important difference.

**Figure S4.** Probability of reduced hospital LOS with PDP+PCR


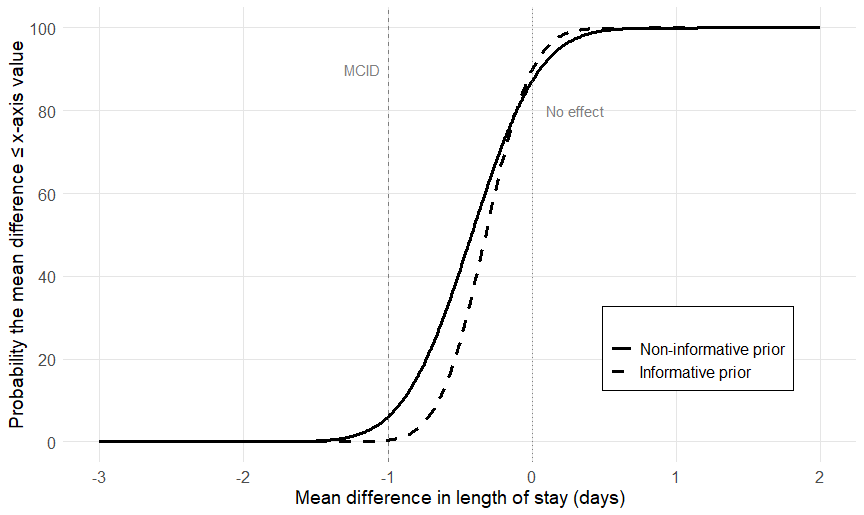


Abbreviations: LOS, length of stay; MCID, minimal clinically important difference; PCR, polymerase chain reaction; PDP, pharmacist-driven protocol.

**Figure S5.** Bayesian posterior probability of hospital readmission reduction with PDP+PCR


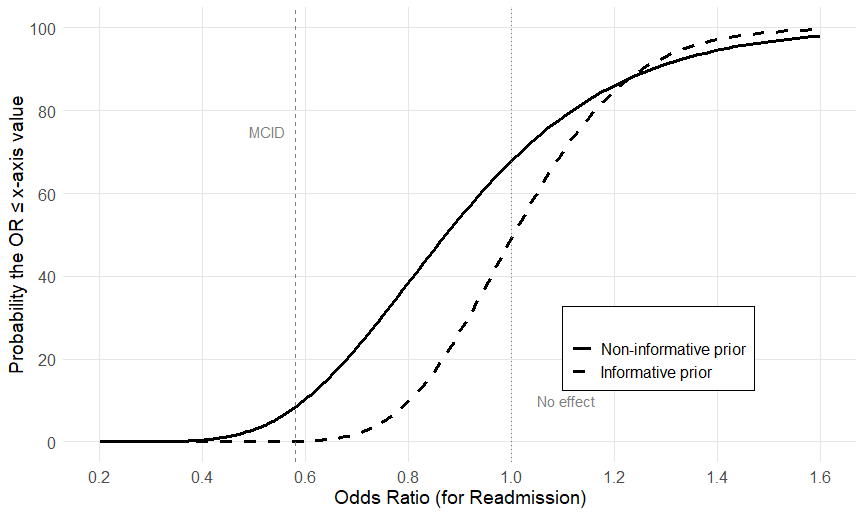


Abbreviations: MCID, minimal clinically important difference; PCR, polymerase chain reaction; PDP, pharmacist-driven protocol.

**Figure S6.** In-hospital mortality cumulative density function of the probabilities plotted against the distribution of effects


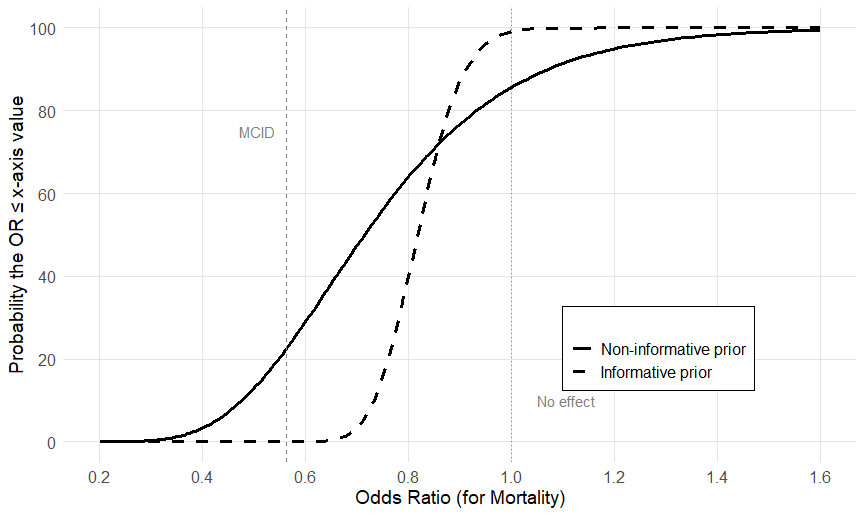


Abbreviations: MCID, minimal clinically important difference.

**Figure S7.** Risk of bias assessments

**
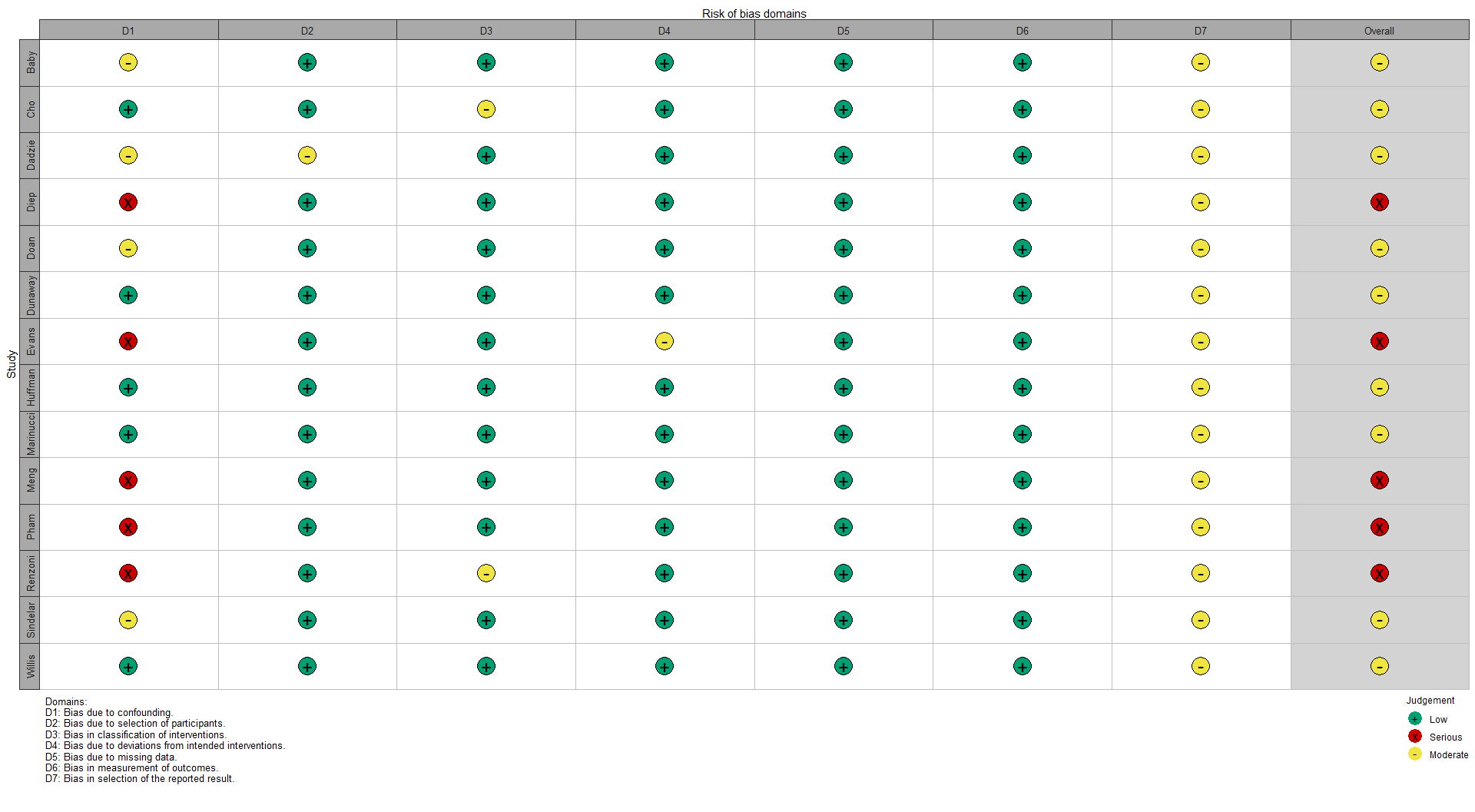
**

**Figure S8**. Funnel plot of days of MRSA therapy with PDP+PCR vs SOC in LRTI patients


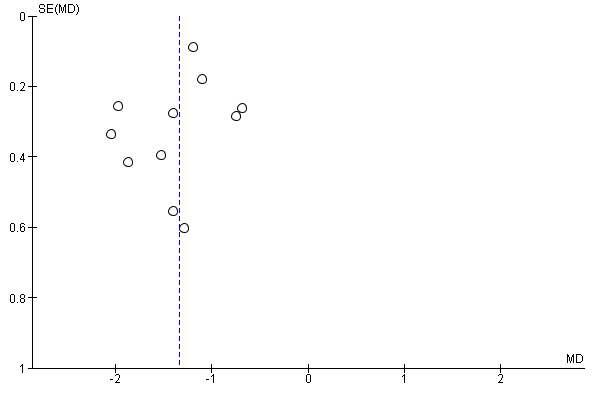


Abbreviations: LRTI, lower respiratory tract infection; MD, mean difference; MRSA, methicillin-resistant

*Staphylococcus aureus*; PCR, polymerase chain reaction; PDP, pharmacist-driven protocol; SE, standard error;

SOC, standard of care.

**Figure S9**. Funnel plot of effect of PDP+PCR on the proportion of patients requiring vancomycin trough monitoring


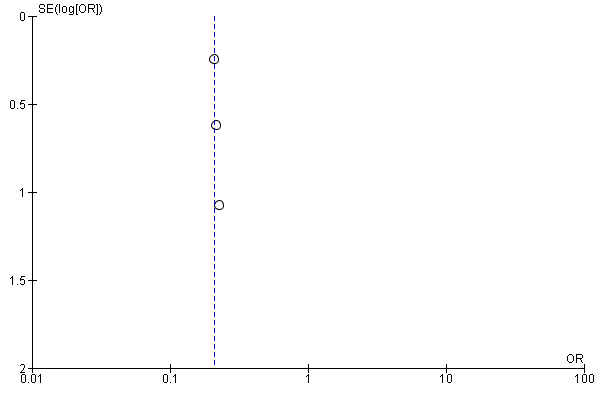


Abbreviations: OR, odds ratio; PCR, polymerase chain reaction; PDP, pharmacist-driven protocol; SE,

standard error.

**Figure S10.** Funnel plot of AKI incidence with PDP+PCR vs SOC in LRTI patients


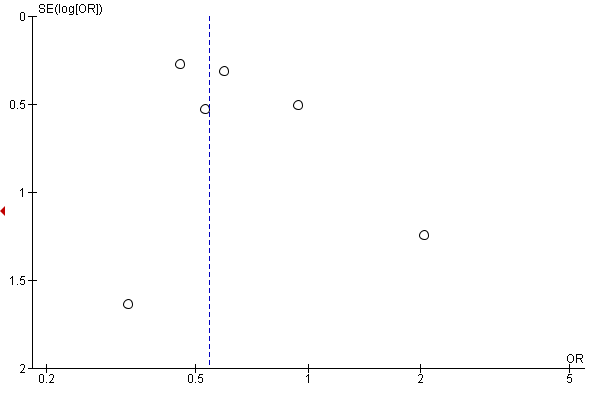


Abbreviations: AKI, acute kidney injury; LRTI, lower respiratory tract infection; OR, odds ratio; PCR,

polymerase chain reaction; PDP, pharmacist-driven protocol; SE, standard error; SOC, standard of care.

**Figure S11.** Funnel plot of hospital LOS with PDP+PCR vs SOC in LRTI patients


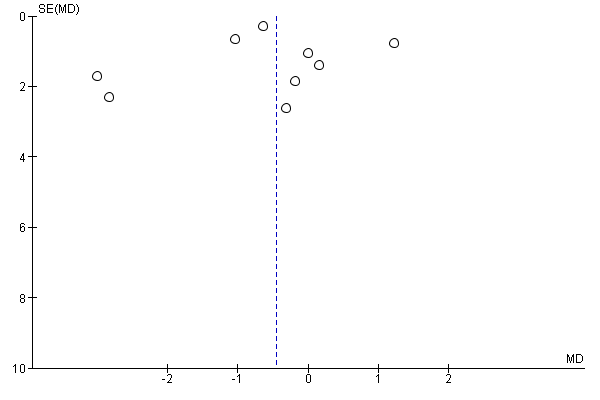


Abbreviations: LRTI, lower respiratory tract infection; MD, mean difference; PCR, polymerase chain reaction;

PDP, pharmacist-driven protocol; SE, standard error; SOC, standard of care.

**Figure S12.** Hospital 30-day readmission rates with PDP+PCR vs SOC in LRTI patients


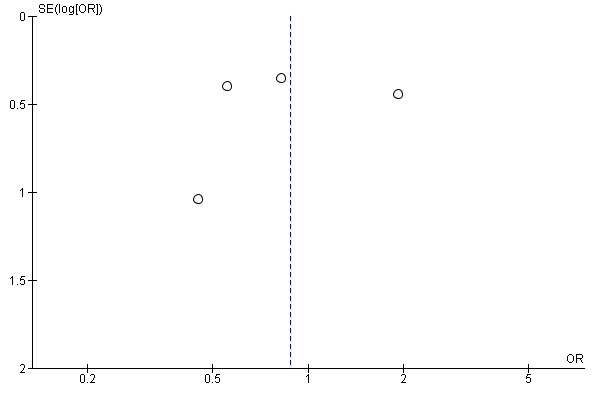


Abbreviations: LRTI, lower respiratory tract infection; OR, odds ratio; PCR, polymerase chain reaction; PDP,

pharmacist-driven protocol; SE, standard error; SOC, standard of care.

**Figure S13.** Funnel plot of in-hospital mortality with PDP+PCR vs SOC in LRTI patients


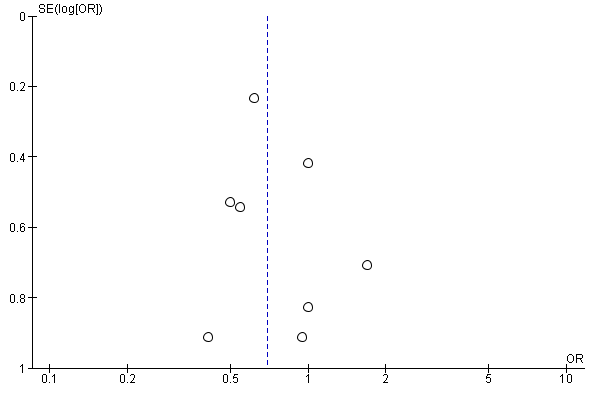


Abbreviations: LRTI, lower respiratory tract infection; OR, odds ratio; PCR, polymerase chain reaction; PDP,

pharmacist-driven protocol; SE, standard error; SOC, standard of care.
